# Ferromagnetism controlled by electric field in tilted phosphorene nanoribbon

**DOI:** 10.1038/srep26300

**Published:** 2016-05-18

**Authors:** M. Umar Farooq, Arqum Hashmi, Jisang Hong

**Affiliations:** 1 Department of Physics, Pukyong National University, Busan 608-737, Korea

## Abstract

Study on phosphorene nanoribbon was mostly focused on zigzag and armchair structures and no ferromagnetic ground state was observed in these systems. Here, we investigated the magnetic property of tilted black phosphorene nanoribbons (TPNRs) affected by an external electric field. We also studied the edge passivation effect on the magnetism and thermal stability of the nanoribbons. The pure TPNR displayed an edge magnetic state, but it disappeared in the edge reconstructed TPNR due to the self-passivation. In addition, we found that the bare TPNR was mechanically unstable because an imaginary vibration mode was obtained. However, the imaginary vibration mode disappeared in the edge passivated TPNRs. No edge magnetism was observed in hydrogen and fluorine passivated TPRNs. In contrast, the oxygen passivated TPNR was more stable than the pure TPNR and the edge-to-edge antiferromagntic (AFM) ground state was obtained. We found that the magnetic ground state could be tuned by the electric field from antiferromagnetic (AFM) to ferromagnetic (FM) ground state. Interestingly, the oxygen passivated TPNR displayed a half-metallic state at a proper electric field in both FM and AFM states. This finding may provoke an intriguing issue for potential spintronics application using the phosphorene nanoribbons.

Recently, a two-dimensional (2D) material so called black phosphorus has drawn considerable attention owing to a successful exfoliation of few-layer black phosphorus named phosphorene^1^,^2^,^3^,^4^ like a graphene. This 2D phosphorene layer has a direct band gap and has also remarkably anisotropic behavior in electrical and optical properties due to its intrinsic puckered layer atomic structure^5^,^6^,^7^,^8^. Numerous studies have investigated various physical properties; for instance, metal adsorption or substitution impurity doping in 2D structures^9^,^10^,^11^,^12^,^13^,^14^. Y. Nakanishi *et al*. performed an experimental work and claimed the existence of magnetism in oxidized pores in black phosphorene layer at room temperature^15^,^16^.

Along with the studies on the 2D structure, the one-dimensional structures such as nanoribbons and nanotubes attract great research interests because of their diverse applications for photophysical, photochemical, and electron transport properties. Particularly, the transport property of nanoribbon is strongly sensitive to the dimensionality because the electronic structure of one-dimentional geometry can be modified by manipulation of their edges^17^,^18^,^19^. Recently, some studies on phosphorene nanoribbons have reported the physical properties regarding the edge passivation^20^,^21^, reconstructions^22^,^23^ and different edge cutting directions^24^. Recently an experimental study has proved that the edge magnetism of zigzag graphene naonribbons can sustain even at room temperature and long range magnetic order can be achieved^25^. Motivated by the edge magnetism in graphene nanoribbons, the edge magnetism in the phosphorene was explored by Z. Zuthe *et al*. in zigzag black phosphorene nanoribbons (ZPNR)^26^. According to their report, the ZPNR displayed magnetic state at the edge atoms if the Peierls distortion did not exist. However, the edge magnetism vanished in the fully relaxed structure. On the other hand, another report on the edge magnetism of ZPNR showed that the edge magnetism still survived even with structure relaxation^27^. Thus, it seems that the edge magnetism of a relaxed structure is still a controversial issue. In our previous investigation, we found that the edge magnetism in the armchair nanoribbons could be obtained by charge doping effect^28^. So far, most of the studies on the phosphorene nanoribbon have been performed with conventional zigzag and armchair direction. Howevr, due to the orthogonal lattice structure of phosphorene, another type of nanoribbon can be obtained by cutting the phosphorene layer at a tilted angle of 54°^29^. Regarding this tilted phosphorene nanoribbon (TPNR), it will be an interesting issue to investigate the magnetism. Therefore, we aim to explore the thermal stability, edge passivation effect, and the edge magnetism of TPNR. Furthermore, we will also investigate the manipulation of magnetic property and electronic structure under an external electric field.

## Results and Discussion

**Figure 1:**
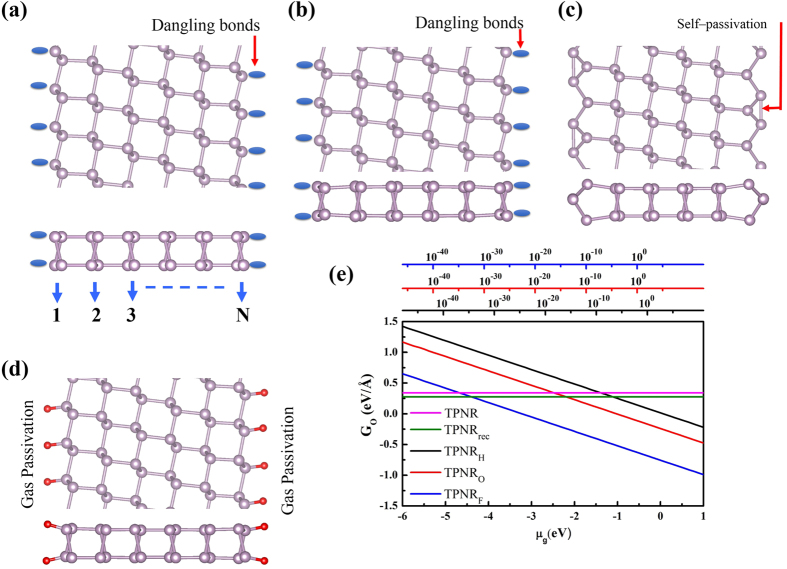
(**a**) Schematic illustration of TPNRs (**b**) relaxed structure of 6-TPNR, (**c**) relaxed structure of 6-TPNR_rec_ (**d**) schematic illustration 6-TPNR with gas atom passivations, red balls represents the H,O and F gas atoms, (**e**) free energies versus chemical potential for 6-TPNR, 6-TPNR_rec_ 6-TPNR_H_ 6-TPNR_O_ and 6-TPNR_F_. The alternative top axes show the pressure, in bar, of molecular gas corresponding to the chemical potentials at room temperature (T = 298.15 K).

**Figure 2:**
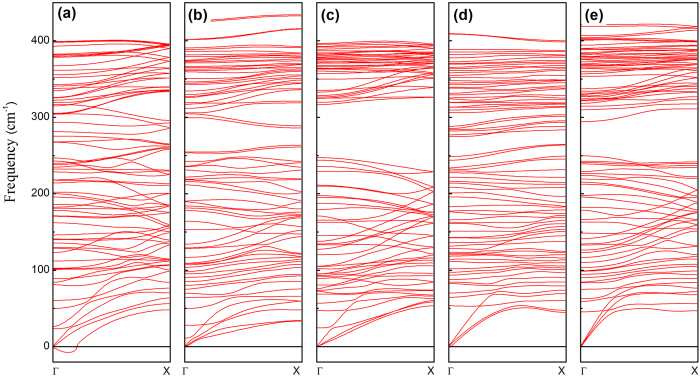
Calculated phonon dispersions of (**a**) 6-TPNR, (**b**) 6-TPNR_rec_ (**c**) 6-TPNR_H_ (**c**) 6-TPNR_O_ and (**d**) 6-TPNR_F_.

Figure 1(a) shows the schematic geometry of this tilted phosphorene nanoribbons (N-TPNRs) in top and side view. In this N-TPNR, two edge atoms per unit cell had dangling bonds. In our study, we considered 6-, 9-, and 12-TPNRs and the corresponding width was 12, 20 and 30 Å. As an illustration, Fig. 1(b) shows the relaxed geometry of 6-TPNR. We found no significant change in the edge structure even after structure optimization and the dangling bond still existed. A recent report on the phosphorene nanoribbons suggests that the bare TPNR may be mechanically unstable because of its large imaginary vibrational modes^29^. However, due to this dangling bond at the edge, there may be another possibility regarding the edge structure because of the edge reconstruction owing to mutual passivation of two dangling bonds. We found that the self-passivated structure in Fig. 1(c) was more stable than the bare relaxed structure in Fig. 1(b). Despite this edge reconstruction, no significant structural modification away from the edge line was observed. We also explored the possibility of edge passivation by hydrogen (H), oxygen (O) and fluorine (F) and their relative stability^17^. Figure 1(d) shows the schematic description for gas passivation and Table 1 shows the calculated edge passivation energies. We observed a lower formation energy in the TPNR_rec_ than the TPNR and this is due to the self-passivation at the edge. In passivated systems, the negative formation energy was obtained. This implies that the edge atom of the TPNR can be naturally passivated if the TPNR exists in the H, O or F rich condition. Among those three types of passivations, the F passivated system has the lowest formation energy. In real experimental conditions, the sample growing will be taking place at finite temperatures. Thus, we calculated the Gibbs free energy per unit edge length (*G*_*o*_) as a function of chemical potential at T = 298 K^30^. Figure 1(e) shows the calculated results. We found that both TPNR_O_ and TPNR_F_ were more stable than TPNR and TPNR_rec_ in oxygen or fluorine gas rich condition at any realistic vacuum pressures. However, the pressure dependency was observed in the TPNR_H_ structure. At low vacuum pressure (>10^−6^ bar), the TPNR_H_ was more stable than both TPNR and TPNR_rec_ in hydrogen gas rich condition. However, the TPNR_H_ became unstable as compared with TPNR and TPNR_rec_ in ultra high vacuum condition. To investigate the mechanical stability of TPNRs, we calculated the phonon frequencies for the 6TPNRs. Figure 2(a) displays the phonon dispersion relation of bare 6TPNR. We observed significant negative frequencies near Γ point. These negative frequencies are the evidence of imaginary vibration mods. These imaginary modes indicate that the bare 6TPNR is unstable. However, we found no negative phonon frequencies in 6TPNR_rec_, 6TPNR_H_, 6TPNR_O_ and 6TPNR_F_ as shown in Fig. 2(b–d). These results exhibit that the passivation leads to a stabilization of the tilted ribbon structure. Indeed, the stabilization of TPNR by hydrogen passivation was recently reported^29^ and our result for hydrogen passivated system agreed with this report.

**Figure 3:**
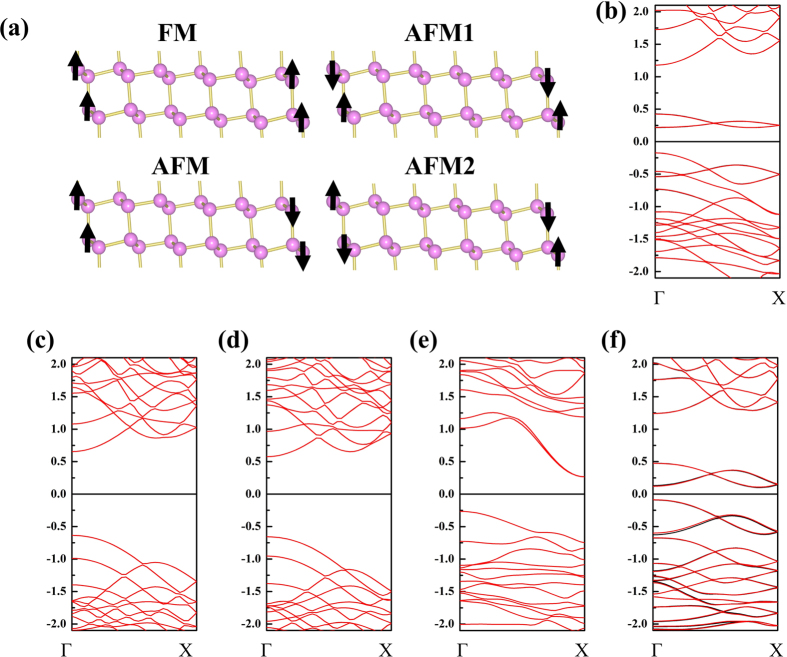
Different spin configurations and calculated band structures of ground states, (**a**) schematic of spin configurations, band structure of (**b**) 6-TPNR, (**c**) 6-TPNR_H_ (**d**) 6-TPNR_F_, (**e**) 6-TPNR_rec_ and (f) 6-TPNR_O_.

Among the five systems shown in Table 1, the magnetic state appeared in TPNR and TPNR_O_ systems. In contrast, the spin polarization at the edge in TPNR_rec_ disappeared due to the self-passivation effect. In addition, we found no magnetic state in H and F passivated nanoribbons. Therefore, we focus on the TPNR and TPNR_O_ systems for further discussion regarding the magnetic properties. We considered four possible spin configurations as displayed in Fig. 3(a). The non-magnetic (NM) state was also considered. In Table 2, we show the total energy differences.

In the TPNR, the AFM was the most stable spin configuration. The calculated magnetic moment at the edge was ~2 μ_B_. However, with increasing the width of the nanoribbon, the total energy difference between FM and AFM almost vanished. In the TPNR_O_ systems, we also found the magnetic moment about 2 μ_B_ at the edge and this is larger than that found in the zigzag phosphorene naonribbons with oxygen passivation (ZPNR_O_)^26^. The total energy difference between FM and AFM is suppressed in TPNR_O_. We now show the band structures. Here it should be noted that we simply used the PBE to describe the exchange and correlation functional (XC). It is well known that the PBE underestimates the band gap while the HSE and GW can match experimental band gap depending on their type^31^. However, the band gap in one dimensional nanoribbon is quite different. For example, previous studies on magnetic zigzag graphene nanoribbons with dangling bonds showed that the conventional XC functional were good enough to describe the edge states in ZNGR^25^,^32^. Here, we chose the 6-TPNR as an illustration. Fig. 3(b) shows the band structure in AFM state. Since the 6-TPNR had an AFM spin configuration, both spin-up and spin-down bands were overlapped and no net magnetic moment appeared. We found edge bands originated from the edge atoms in the top of the valence and low-lying conduction bands near the Fermi level. Figure 3(c,d) shows the band structures of H and F passivated systems. The band structures of nanoribbons with H and F passivation have similar characteristic. In these structures, the edge state disappeared. In the H passivated case, the broken bond at the edge had an electron to form a complete sp^3^ bonding with H atom and the edge state vanished. In the F passivation, the similar behavior took place. Therefore, the band structures in both systems were almost identical. In the TPNR_rec_ in Fig. 3(e), we found an indirect band gap and the edge bands observed in the TPNR disappeared due to the self-passivation effect. Figure 3(f) shows the band structure of oxygen passivated system. Interestingly, the edge bands reappeared in the TPNR_O._ In the oxygen passivation structure, one electron from the oxygen participated in the bond like in the hydrogen passivation case, but another electron contributed to edge induced a magnetic moment. In case of graphene nanoribbon, four valence electrons of single carbon atom are bonded with three other carbon atoms. Therefore, the removal of single bond in zigzag direction created a magnetic moment around 1.28 μ_B_. After passivation with hydrogen, this magnetic moment reduced to 0.26 μ_B_ due to hybridization effect^33^,^34^. Unlike the graphene nanoribbon, the TPNR has two edge atoms on each side and the magnetic moment of 1 μ_B_ per one edge atom was observed and the same magnetic moment was achieved in TPNR_O_ even after hybridization of oxygen atom.

**Figure 4:**
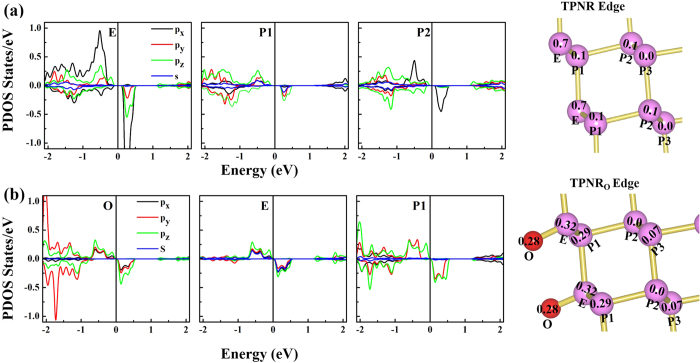
Partial density of state (PDOS) with the magnetic moment of edge atoms (**a**) PDOS for E , P1 and P1 atoms from left to right and magnetic moments of edge atoms with labeling (**b**) PDOS for 6-TPNR_O_ of O , E and P1 atoms from left to right and magnetic moments of edge atoms with labeling.

Figure 4(a) shows the calculated partial density of states (PDOS) and magnetic moment of P atoms near the edge in the TPNR. The value inside a circle represents the magnetic moment of a specific atom. In the TPNR, the magnetic moment was mostly localized at the edge atom indicated by “E” while the nearest neighboring P atom (P1) or another P atom (P2) next to P1 had very small magnetic moment. The TPNRs can be obtained by removing a single in-plane bond in the edge atom and this removal of the bond resulted in the magnetism dominated by the p_x_ orbital at the edge while we observed a relatively weak spin polarization in the next to the edge atom. Figure 4(b) shows the PDOS and the magnetic moment of edge atoms for the TPNR_O_. In the ZPNR_O_, the local magnetic moments of 0.27 μ_B_ for O atom, 0.39 μ_B_ for edge phosphorous atom and 0.13 μ_B_ for next to edge atom was reported^26^. Here in TPNR_O_, the magnetic moments were equally distributed over O, E edge phosphorous, and P1 next to edge atoms. In the oxygen atom, all the three p orbitals almost equally contributed to the magnetic moment, due to the orbital hybridization between the edge phosphorous (E) and oxygen atoms. We found that the spin asymmetric PDOS in E atom was somewhat suppressed while it was enhanced in P1 atom. As a result, all three atoms preserved almost the same magnetic moment. In contrast, the ZPNR was obtained by cutting the single out of plain bond. This creates a dangling bond dominated by the p_z_ orbital. After the oxygen passivation, the p_z_ orbitals from O and edge P atoms mostly contributed to the spin polarized state^26^.

**Figure 5:**
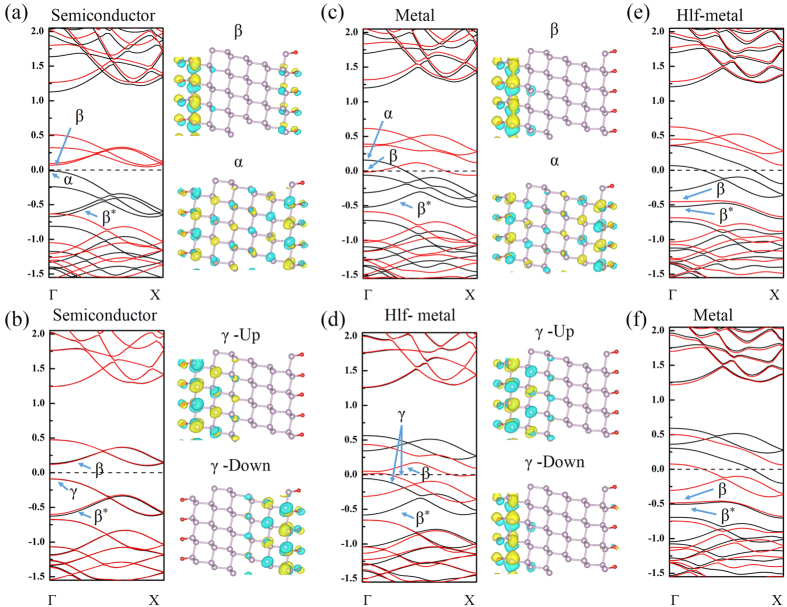
Calculated band structures and wave functions of labeled bands for TPNR_O_ at different electric field strengths, (**a**) for FM at zero field, (**b**) for AFM at zero field, (**c**) for FM at 0.3 V/Å, (**d**) for AFM at 0.3 V/Å, (**e**) for FM at 0.4 V/Å and (**f**) for AFM at 0.4 V/Å. The wave functions are at Γ point with same iso-surface. In band structure, black lines represents spin-up and red lines spin-down channels.

**Figure 6:**
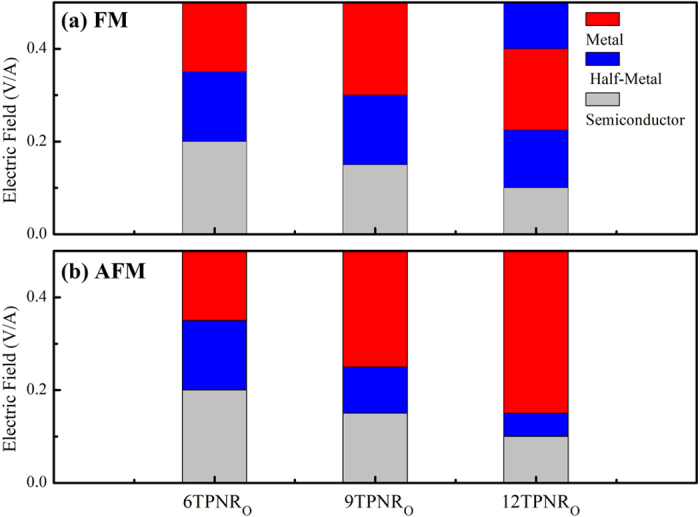
Stack columns indicating the band characters of TPNR_O_ at different electric field strengths in (**a**) FM (**b**) AFM spin configurations.

**Figure 7:**
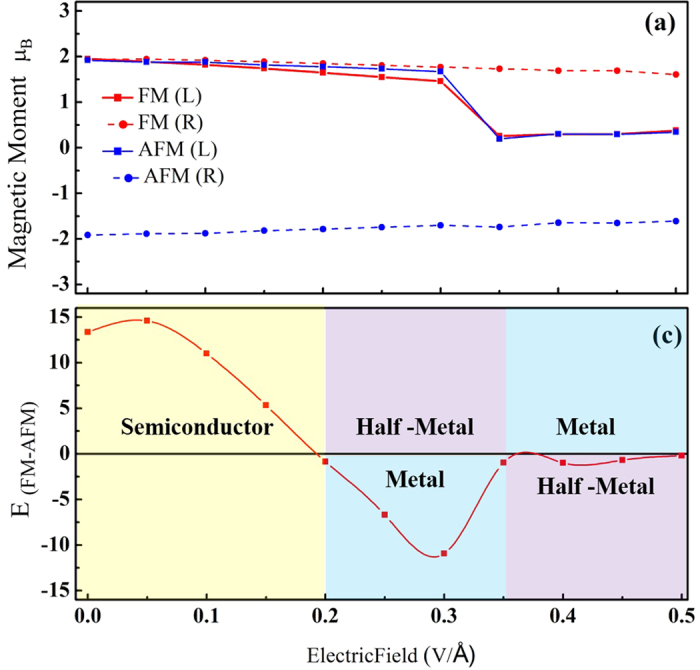
(**a**) For the magnetic moment on the left and right edges and (**b**) the energy difference FM and AFM with the change of electric field.

We now discuss the electric filed effect on the magnetic property. Since the TPNR is mechanically unstable, we focus on the TPNR_O_. Figure 5(a–f) shows the band structures of 6-TPNR_O_ in both FM and AFM states with an increasing electric field. Here, we assumed that the electric field is applied from the left to the right direction of the TPNR. With the increase of applied electric field, the electrons moved to the left edge. As a result, the states originated from the left edge atoms shifted downward while the right edge atoms contributed to the opposite behavior. Consequently, the unoccupied states from the left edge and occupied state from the right edge moved close to the Fermi level. This feature results in the splitting of the overlapping two spin bands in AFM state and eventually forces the crossing of Fermi level of spin-down band to make it half-metallic regarding the transport property even in an anti-ferromagnetic state. The onset of this feature was observed at 0.2 V/Å. On the other hand, for the FM configuration, the band structure showed an ordinary metallic behavior at the same electric field. With further increasing the electric field, the partially field electron state become completely field. This leads to the complete rearrangement of charge at the edge and magnetic moment on left edge quenches. At the field strength of 0.35 V/Å and higher, the band structure was significantly changed. In Fig. 5(e–f) the β and β^*^ bands belonged to the left edge were overlapped. On the other hand, the occupied states originated from the right edge become partially unoccupied. However, no sudden rearrangement of charge occurred on left edge. After the field strength of 0.35 V/Å, the FM state showed a half-metallic behavior while the AFM state displayed an ordinary metallic feature. For 9-TPNR and 12-TPNR, we observed the similar band characteristics although the energy difference E_FM-AFM_ was almost zero. Figure 6 summarizes the band characteristics of all the systems at different electrified strengths. Figure 7(a) displays the magnetic moment at both edges with the change in the electric field for FM and AFM configurations. The magnetic moment gradually decreased. However, we found a sharp decline in the magnetic moment at the left edge at 0.35 V/Å and the magnetic moment was about 0.3 μ_B_. On the other hand, the right edge had a magnetic moment about 1.7 μ_B._ Therefore, the ribbon showed a ferrimagnetic state in AFM configuration. Along with the change in the band structures, the applied electric filed also affected the magnetic ground state. Figure 7(b) shows the total energy difference between FM and AFM states. As displayed, we found an oscillatory behavior in the total energy difference. As described in the band structures in Fig. 5, the TPNR_O_ had a band gap in both FM and AFM states. The AFM ground state with a semiconducting band gap was sustained up to a certain field, but the energy difference was decreased with the electric field. When the AFM state displayed a metallic behavior, the magnetic ground state changed from AFM to FM. The experimental band gap of TPNR or TPNR_O_ is not yet available for comparison. We remark that the experimental band gap may be different from our values presented in this report. The different gap may require different electric field strength to change from semiconducting to metallic state. However, the switching from AFM to FM takes place only when the band structure shows metallic behavior. Therefore, in the presence of metallic state, our results can explain the essential physics of magnetic switching. Beyond the field strength of 0.35 V/Å, we found no meaningful total energy difference. In a semi-qualitative manner, we interpret the results in this way. If two spins at both edges have an anti-parallel configuration, the overall spatial wavefunction should be symmetric and this increases the Coulomb repulsive energy. On the other hand, the parallel spin configuration has an anti-symmetric spatial wavefunction and this causes relatively weaker Coulomb repulsive energy. However, the kinetic energy in symmetric wavefunction is smaller than the anti-symmetric case because the kinetic energy is related to the curvature of the wavefunction. In the 6-TPNR_O_ at zero field, the AFM was more stable than the FM. This implies that the kinetic energy term is a dominant factor for determining the ground state. With increasing the electric field, the transition from a semiconducting to a metallic state was found. Consequently, the relative importance of the Coulomb repulsive interaction was increased. Due to this feature, the FM became a ground state. As a result, the magnetic interaction between two edges was suppressed and this caused the reduction in total energy difference. To explore this in more details, we investigated the wavefunctions belonging to the edge states near the Fermi-level and the wavefunctions corresponding to the edge states are presented in Fig. 5. For FM states at zero field, α represents the valence band maximum which has only a spin-up component in the occupied states and β represents conduction band minimum which has only a spin-down component in the unoccupied states. For AFM state at zero field, the valence band maximum labeled as γ which has both spin-up and -down components in occupied states. In FM configuration at zero electric field, the tail of the wavefunction in the central layer was found while no such tail was observed in AFM state. This suggests that the kinetic energy in FM configuration was larger than in AFM state. At 0.3 V/Å, the wavefunction characteristics of α and β states in FM state were slightly changed. However, in AFM state, we found that the spin-down electrons of the γ state shifted to the left edge while the spin-up electrons already existed on the left edge. Thus, both spin up and down electrons existed in the left edge and this caused a strong Coulomb repulsive interaction. As a result, the total energy was increased and the FM state became a ground state. Further increasing the electric field, we observed that the magnetic moment in the left edge was greatly suppressed. Consequently, the energy difference almost vanished. For 9TPNR_O_, the edge-to-edge distance was already too large to interact each other. Therefore, the total energy difference is very small and it is meaningless to distinguish it.

## Discussion

In summary, we investigated the structural and magnetic properties of TPNRs including the edge passivation effect. The pure TPNR displayed a magnetic state, but the edge reconstruction eliminated the edge magnetism due to the self-passivation. Besides, we found no magnetic state in TPNR_H_ and TPNR_F_. Interestingly, the magnetic state was still found in TPNR_O_ and it is stable in oxygen rich condition. The In TPNR and TPNR_O_, the edge-to-edge coupling had an AFM interaction in the ground state with a semiconducting band gap although the total energy difference was dependent on the width of the nanoribbons. The transition from a semiconductor to a metallic state was found with increasing the electric field strength in both FM and AFM configurations. Besides, the electric filed induced an oscillatory behavior in the total energy difference between the ferromagnetic to antiferromagnetic state in the TPNR_O_. Beyond the field strength of 0.35 V/Å, no meaningful total energy difference was observed due to the suppression of magnetic moment from the one side of nanoribbon. More interestingly, we found that the TPNR_O_ displayed half metallic characteristic in both FM and AFM at a proper electric filed and this may provoke an interesting issue for potential application for spintronics.

### Computational Methods

We performed the first principles calculations by using Spanish Initiative for Electronic Simulations with Thousands of Atoms (SIESTA) method^35^. Many-body effects were described within the generalized gradient approximation (GGA) by applying the Perdew−Burke−Ernzerhof (PBE) exchange-correlation functional^36^. We used Troullier–Martins norm-conserving pseudopotential for the treatment of core electrons. Variationally optimized double-ζ plus polarized (DZP) basis sets were used to simulate the valence electron^37^,^38^. Brillouin zone was sampled by 46 k-points using Monkhorst–Pack k-grid scheme^39^. Real space integrations were performed on a mesh with an energy cut-off of 400 Ry. The energy convergence criterion was kept 10^−5^ eV for electronic structure calculation. To investigate the electric field dependency, we applied in-plane homogeneous electric fields across the edges of the TPNRs and the atomic structure is relaxed until the force on each atom reached below the 0.01 eV/Å. We also calculated the phonon dispersion curve to check the mechanical stability of the TPRN and a 3 × 1 × 1supercell was used.

## Additional Information

**How to cite this article**: Farooq, M. U. *et al*. Ferromagnetism controlled by electric field in tilted phosphorene nanoribbon. *Sci. Rep*. **6**, 26300; doi: 10.1038/srep26300 (2016).

## Figures and Tables

**Table 1:** Edge formation energy *ε*_*edge*_ (in eV) for different edges.

Widths/Edge type	6-TPNR	9-TPNR	12-TPNR
TPNR	0.349	0.345	0.340
TPNR_rec_	0.284	0.279	0.274
TPNR_H_	−0.005	−0.010	−0.015
TPNR_O_	−0.227	−0.233	−0.237
TPNR_F_	−0.755	−0.760	−0.764

**Table 2:** Total energy differences (in meV/cell) for different magnetic configurations of TPNR and TPNR_O_.

Ribbon	E_NM-FM_	E_FM-AFM_	E_AFM-AFM1_	E_AFM1-AFM2_
6-TPNR	506.91	20.94	−68.10	−21.45
9-TPNR	590.89	1.22	−69.65	1.02
12-TPNR	597.02	0.20	−68.43	−0.44
6-TPNR_O_	264.70	13.34	−15.16	1.22
9-TPNR_O_	269.00	1.20	−13.98	0.17
12-TPNR_O_	262.90	−0.10	−14.49	0.00
